# TACC3–ch-TOG interaction regulates spindle microtubule assembly by controlling centrosomal recruitment of γ-TuRC

**DOI:** 10.1042/BSR20221882

**Published:** 2023-03-16

**Authors:** Resmi Rajeev, Swarnendu Mukhopadhyay, Suresh Bhagyanath, Manu Rani S. Devu Priya, Tapas K. Manna

**Affiliations:** School of Biology, Indian Institute of Science Education and Research Thiruvananthapuram, Thiruvananthapuram 695551, India

**Keywords:** centrosome, microtubule, mitosis, mitotic spindle, TACC3

## Abstract

γ-Tubulin ring complex (γ-TuRC), composed of γ-tubulin and multiple γ-tubulin complex proteins (GCPs), serves as the major microtubule nucleating complex in animal cells. However, several γ-TuRC-associated proteins have been shown to control its function. Centrosomal adaptor protein, TACC3, is one such γ-TuRC-interacting factor that is essential for proper mitotic spindle assembly across organisms. ch-TOG is another microtubule assembly promoting protein, which interacts with TACC3 and cooperates in mitotic spindle assembly. However, the mechanism how TACC3–ch-TOG interaction regulates microtubule assembly and the γ-TuRC functions at the centrosomes remain unclear. Here, we show that deletion of the ch-TOG-binding region in TACC3 enhances recruitment of the γ-TuRC proteins to centrosomes and aggravates spindle microtubule assembly in human cells. Loss of TACC3–ch-TOG binding imparts stabilization on TACC3 interaction with the γ-TuRC proteins and it does so by stimulating TACC3 phosphorylation and thereby enhancing phospho-TACC3 recruitment to the centrosomes. We also show that localization of ch-TOG at the centrosomes is substantially reduced and the same on the spindle microtubules is increased in its TACC3-unbound condition. Additional results reveal that ch-TOG depletion stimulates γ-tubulin localization on the spindles without significantly affecting the centrosomal γ-tubulin level. The results indicate that ch-TOG binding to TACC3 controls TACC3 phosphorylation and TACC3-mediated stabilization of the γ-TuRCs at the centrosomes. They also implicate that the spatio-temporal control of TACC3 phosphorylation via ch-TOG-binding ensures mitotic spindle assembly to the optimal level.

## Introduction

The centrosome is the primary microtubule nucleating center in animal cells and its aberrations are linked to multiple diseases including cancer and developmental disorders [1,2]. Centrosome comprises a pair of centrioles embedded in the pericentriolar material (PCM), which orchestrates assembly and organization of the mitotic spindles by constituting the microtubule-organizing center (MTOC). Optimal function of the MTOC requires recruitment of many structural and regulatory proteins to the PCM, which activate microtubule nucleation and assembly. Critical among those are γ-tubulin and its associated protein complexes that are recruited to the PCM in the form of large ring complexes, which provide the structural platform to nucleate microtubules from the MTOC. Specifically, γ-tubulin and conserved γ-tubulin-binding proteins/complex proteins, GCP2 and GCP3, form a sub-complex called γ-tubulin-small complex (γ-TuSC). Multiple such γ-TuSCs together with additional GCP proteins, such as GCP 4, 5, and 6, and other interacting proteins, such as MZT family proteins and CDK5RAP2 (CDK5 regulatory subunit-associated protein 2) assemble to constitute the larger Gamma-tubulin ring complex (γ-TuRC) [3–6]. As cells enter the M phase, the centrosomal recruitment of γ-tubulin ring complex proteins increases ∼3- to 5-folds, resulting in increased microtubules density at the centrosomes [7,8]. Although the γ-TuRC serves as the main microtubule nucleation machinery, several other factors are required for their activation and optimal regulation of its microtubule nucleation ability [9–12]. Previous studies have demonstrated that the purified γ-TuRCs exhibit very weak microtubule nucleation activity as the structural organization of the γ-TuRC is not structurally compatible to initiate microtubule assembly using the α,ß-tubulin dimers [13–16]. It suggests the involvement of other cooperating proteins for regulation of the microtubule nucleation by the γ-TuRCs in cells. Molecular mechanisms underlying regulation of centrosomal microtubule nucleation by the γ-TuRCs remain to be fully understood.

Recent studies have identified important roles of the centrosomal coiled-coil protein, TACC (transforming acidic coiled-coil) in microtubule nucleation and in regulation of γ-TuRC stabilization at the centrosomes. Immuno-depletion of Maskin/TACC3 in *Xenopus laevis* or siRNA-mediated TACC3 depletion in human cells results in loss of centrosomal asters [10,17]. Studies in human cells showed that TACC3 is required for localizing the components of the γ-TuRC to the centrosomes in mitotic cells [10]. Moreover, loss of TACC3 affects the assembly of γ-TuRCs from the γ-tubulin small complex components (γ-TuSCs) in human cell lysates, indicating that TACC3 is involved in stabilizing the ring complex [10]. Additionally, TACC3-mediated centrosomal microtubule assembly and its γ-TuRC-stabilizing activities are dependent on its phosphorylation at a conserved Aurora A-targeting site [18]. XMAP215, *Xenopus* homolog of human ch-TOG, has emerged as another key regulator of microtubule polymerization and nucleation in recent years [11,19–21]. XMAP215/ch-TOG is a multi-domain protein consisting of five TOG domains in the N-terminus and a C-terminal domain [20]. The TOG domains bind to α,β-tubulin dimers and are majorly responsible for the microtubule polymerization-promoting activity of XMAP215/ch-TOG [22,23]. Based on studies of XMAP215 of *Drosophila*, Msps, XMAP215 was also speculated to have a sixth TOG in its C-terminus [24–26]. While the TOG domains [1–5] of XMAP215 are responsible for microtubule polymerization by catalyzing addition of α,β-tubulin dimers, its C-terminus has been shown to stimulate microtubule nucleation synergistically with the γ-TuRC in *Xenopus* egg extracts [11,22,23]. The purified form of XMAP215 C-terminus can also bind to γ-tubulin *in vitro*, though the role of that interaction in centrosomal microtubule nucleation *in vivo* is not known [11].

Homologs of TACC3 and ch-TOG exhibit evolutionary conserved interactions that are linked to regulation of centrosomal microtubule functions [27–29]. In *Drosophila*, D-TACC recruits Msps (Minispindles/ch-TOG) to the centrosomes and promotes centrosomal microtubule stability [28,30]. Deletion/deletion of TACC3 impairs centrosomal localization of ch-TOG/XMAP215 in diverse organisms and cell types [28,30–33]. In human somatic cells, ch-TOG–TACC3 interaction appears to play critical role in organizing the spindle poles and apparently, also a minor role in stabilizing the spindle microtubules [34]. However, both the proteins also exhibit some levels of centrosomal localization independent of each other [27,34–36]. The coiled-coil domain of TACC3 in its TACC domain interacts directly with the C-terminal [1517–1957] region of ch-TOG via a 3-aa break/stutter region [24,37]. The binding region in ch-TOG was later identified as the 1932–1957 aa region in the C-terminus of ch-TOG, right after the putative TOG6 domain at position 1517–1802 aa [24–26,38,39]. TACC3 mutation by deleting of either of the break regions, (Δ678–681 and Δ682–688) abolished TACC3–ch-TOG interaction; though it neither affected the overall structure of the TACC domain; nor it affected the spindle localization of TACC3 [24]. Though TACC3's role in centrosomal recruitment of the γ-TuRC and that of ch-TOG–TACC3 interaction on spindle microtubule stability have been demonstrated, the mechanisms how TACC3–ch-TOG interaction contributes to centrosomal microtubule nucleation and the centrosomal recruitment of γ-TuRC in human cells have remained unclear.

Here, we show that the abrogation of TACC3–ch-TOG interaction leads to increased recruitment of the γ-TuRC proteins to the centrosomes and thereby results in increased microtubule assembly at the mitotic centrosomes in human cells. TACC3 interaction with the γ-TuRC proteins is hyper-stabilized under such condition and further, ch-TOG localization to the centrosomes is diminished with concomitant increase of its localization on the mitotic spindles. Interestingly, inhibition of TACC3–ch-TOG interaction promotes TACC3 phosphorylation at its Aurora A-targeting Ser 558 site and thereby stimulates centrosomal localization of Ser558-phosphorylated TACC3, which results in increased stabilization on TACC3–γ-TuRC interaction. The results indicate that TACC3–ch-TOG interaction controls the levels of phosphorylated TACC3 and its-mediated γ-TuRC recruitment to centrosomes and such mechanism ensures the spindle microtubule assembly to the required level.

## Results

### Perturbation of TACC3–ch-TOG interaction stimulates recruitment of γ-TuRC proteins to centrosomes

**Figure 1:**
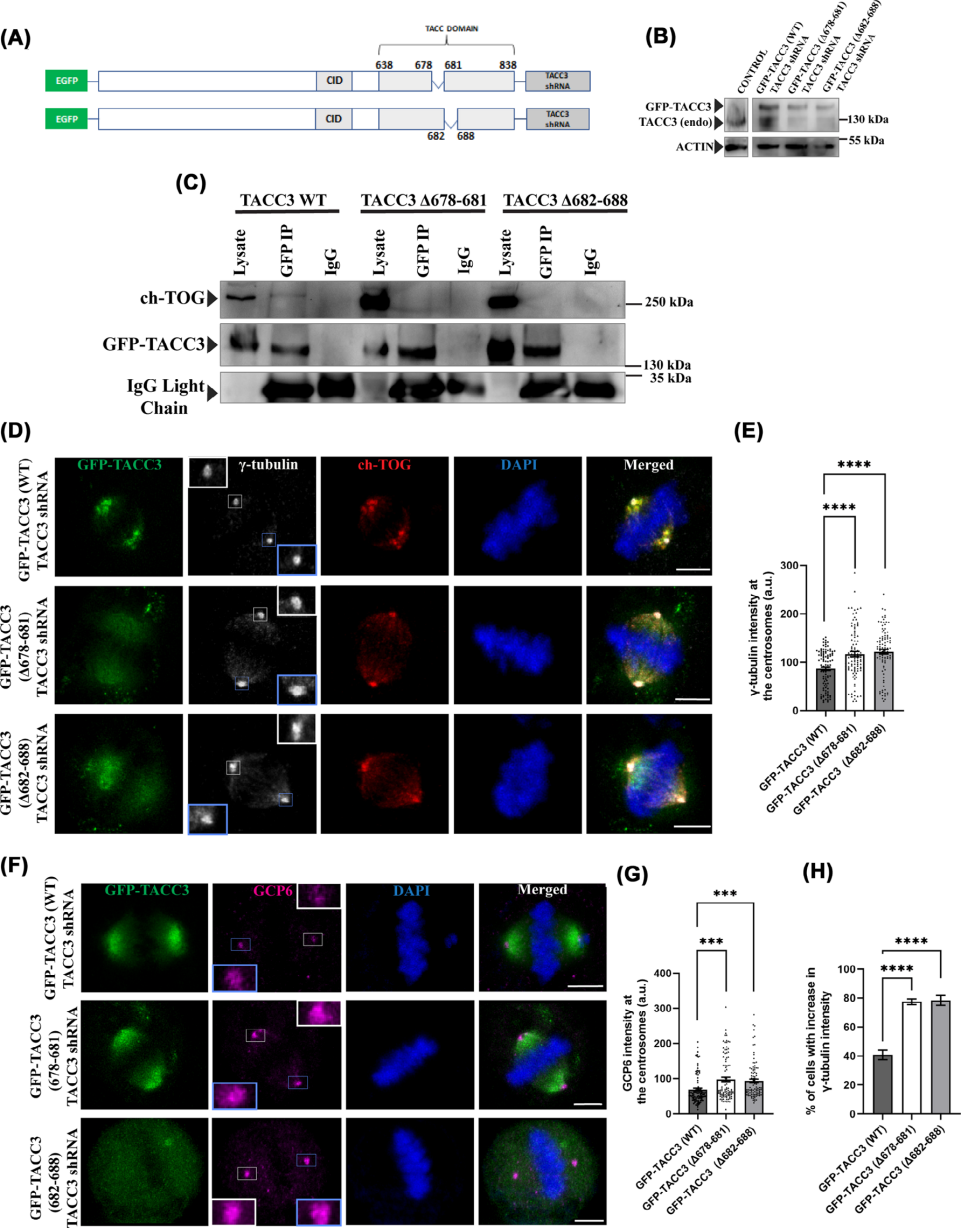
Perturbation of TACC3–ch-TOG interaction stimulates recruitment of γ-TuRC proteins to centrosomes (**A**) Schematic representation of GFP-TACC3 (Δ678–681)-TACC3 shRNA and GFP-TACC3 (Δ682–688)-TACC3 shRNA constructs. (**B**) Lysates of HeLa Kyoto cells transfected with GFP-TACC3 (WT)-TACC3 shRNA, GFP-TACC3 (Δ682–688)-TACC3 shRNA, or GFP-TACC3 (Δ678–681)-TACC3 shRNA for 48 h were analyzed for exogenous TACC3 proteins with simultaneous depletion of endogenous TACC3. Both endogenous TACC3 and GFP-TACC3 proteins were stained with mouse monoclonal TACC3 antibody. Actin was probed as a control. TACC3 level in control untrasfected cells is also shown. (**C**) Immunoprecipitation of GFP-tagged TACC3 proteins using the anti-GFP antibody in the lysates of HeLa Kyoto cells transfected with GFP-TACC3 (WT)-TACC3 shRNA, GFP-TACC3 (Δ678–681)-TACC3 shRNA, or GFP-TACC3 (Δ682–688)-TACC3 shRNA. The immunoblots were probed with GFP and ch-TOG antibodies. (**D**) Representative confocal images of GFP-TACC3 (WT)-TACC3 shRNA (top panel), GFP-TACC3 (Δ678-681)-TACC3 shRNA (middle panel), or GFP-TACC3 (Δ682–688)-TACC3 shRNA (bottom panel)- transfected mitotic HeLa Kyoto cells showing the differences in γ-tubulin levels at the centrosomes. Scale bar, 5 μm, γ-tubulin, and ch-TOG were stained with mouse monoclonal anti-γ-tubulin and rabbit polyclonal anti-ch-TOG antibody, respectively. (**E**) The bar graph shows the quantification of γ-tubulin intensity at the centrosomes (two) in different conditions as of (d). (**F**) Representative confocal images of GFP-TACC3 (WT)-TACC3 shRNA (top panel), GFP-TACC3 (Δ678–681)-TACC3 shRNA (middle panel), or GFP-TACC3 (Δ682–688)-TACC3 shRNA (bottom panel) transfected mitotic HeLa Kyoto cells showing the differences in GCP6 localization at centrosomes. Scale bar, 5 μm, GCP6 was stained with mouse monoclonal anti-GCP6 antibody. (**G**) The bar graph shows the quantification of GCP6 intensity at the centrosomes in different conditions as in panel (F). (**H**) The bar graph shows the percentage of cells (cells with above the average centrosomal γ-tubulin intensity of TACC3 WT-expressed cells) with increased γ-tubulin intensity phenotype in both the mutant cases. The bars in (e), (g), and (h) represent mean ± S.E. The number of mitotic cells counted = 100 each (four independent experiments); ****, *P*<0.0001, ***, *P*<0.001.

We first aimed to determine the exclusive role of TACC3 independent of its interaction with ch-TOG on the centrosomal regulation of γ-TuRC and microtubule assembly at the centrosomes and it was assessed by disrupting TACC3–ch-TOG interaction by expressing TACC3 deletion mutants, TACC3 Δ678–681 and TACC3 Δ682–688, that fail to bind ch-TOG. Deletion of either of these amino acid stretches in TACC3 has been shown to disrupt TACC3 interaction with ch-TOG (Figure 1A) [24,38]. HeLa Kyoto cells were transfected with fusion plasmids consisting of shRNA resistant TACC3 Δ678–681 or TACC3 Δ682–688 and TACC3 shRNA, named as GFP-TACC3 (Δ678–681)-TACC3 shRNA and GFP-TACC3 (Δ682–688)-TACC3 shRNA, respectively, (also denoted as TACC3 deletion mutants) and then treated with thymidine and released thereafter (Materials and methods) for mitotic synchronization (Figure 1A). Transfection of the plasmids enabled expression of the TACC3 deletion mutants with simultaneous depletion of endogenous TACC3 by the TACC3 shRNA present in the constructs (Figure 1B). Expression of the GFP-TACC3 (WT) was also checked by transfecting the cells with GFP-TACC3 (WT)-TACC3 shRNA, referred as TACC3-WT elsewhere (Figure 1B), which was also TACC3 sh-RNA resistant [18,24]. Consistent with earlier studies, it was confirmed by co-immunoprecipitation (co-IP) using GFP antibody that both the TACC3 deletion mutants failed to interact with the endogenous ch-TOG, while ch-TOG showed association with the GFP-TACC3 WT (Figure 1C). These analyses confirmed that ch-TOG-binding ability was effectively abrogated for both the TACC3 deletion mutants. Next, we investigated how the abrogation of TACC3–ch-TOG binding affects centrosomal localization of the γ-TuRC proteins. HeLa Kyoto cells expressed with the TACC3 deletion mutants were immunostained for the γ-TuRC proteins, γ-tubulin, and GCP6, two key components of the ring complex. Mitotic synchronized cells expressed with either of the TACC3 deletion mutants showed increased localization of γ-tubulin at the centrosomes as compared with the TACC3-WT-expressed cells (Figure 1D). Intensity analysis of γ-tubulin showed a ∼40% increase in its centrosomal localization in both the deletion mutants compared with the wild-type expressed cells (Figure 1E) and the extents of increase were quite significant statistically. A similar increase was observed for the centrosomal localization of GCP6 (Figure 1F,G). Approximately 80% of cells displayed the increased γ-tubulin intensity phenotype in both the mutant cases (Figure 1H). Though the images of individual cells in some cases showed slight variations of GFP levels visibly, which happened to be observed both in the case of TACC3-WT and TACC3-mutants conditions, but the increased centrosomal localizations of γ-tubulin and GCP6 were apparent in majority of the mutants-expressed cells as compared with the TACC3-WT cells. These results indicate that TACC3–ch-TOG binding controls centrosomal recruitment of the γ-TuRC proteins.

### Abrogation of TACC3–ch-TOG interaction leads to abnormally dense spindle microtubules

**Figure 2:**
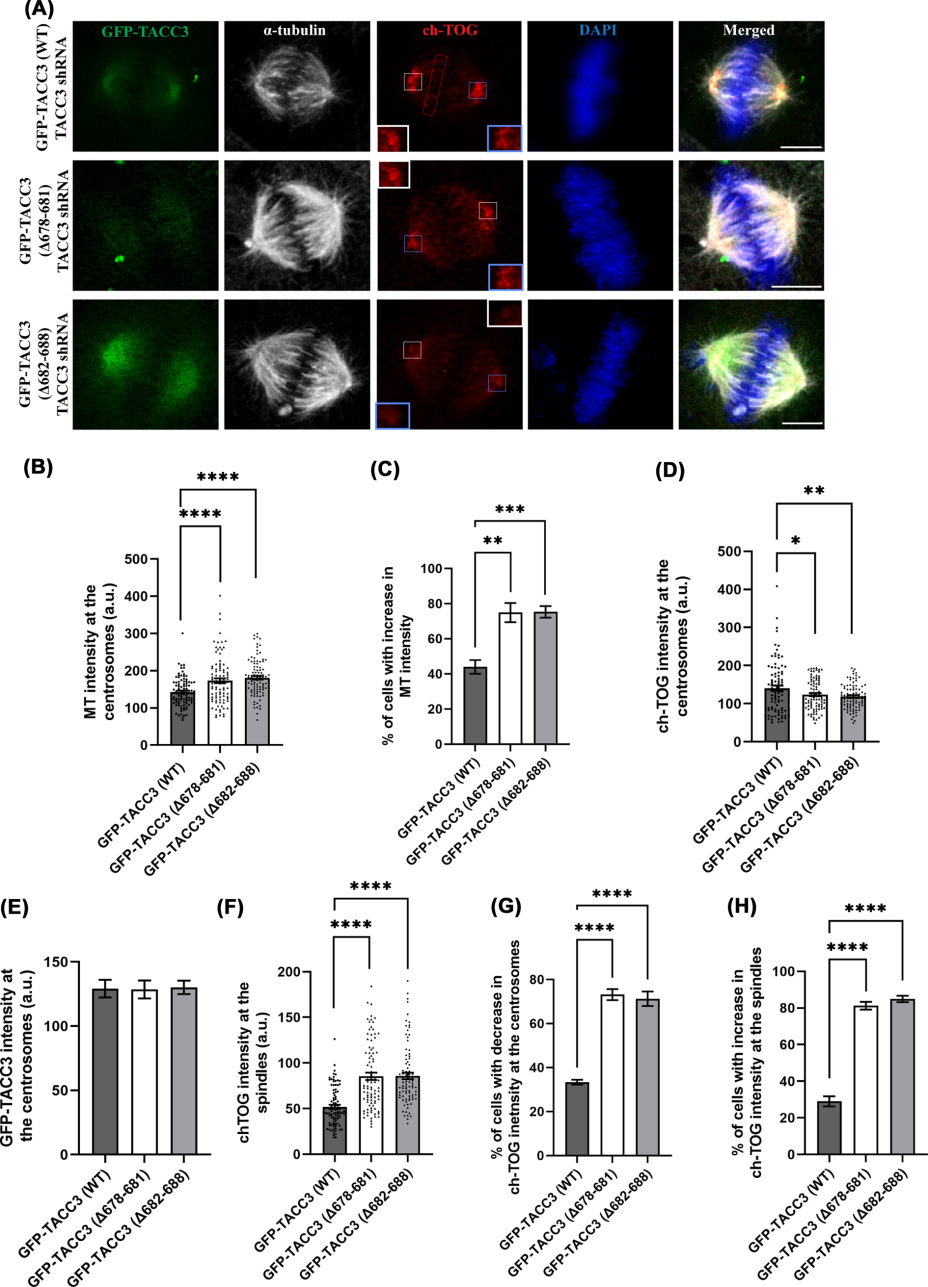
Abrogation of TACC3–ch-TOG interaction leads to abnormally dense spindle microtubules (**A**) Representative confocal images of GFP-TACC3 (WT)-TACC3 shRNA (top panel), GFP-TACC3 (Δ678–681)-TACC3 shRNA (middle panel), or GFP-TACC3 (Δ682–688)-TACC3 shRNA (bottom panel) transfected mitotic HeLa Kyoto cells showing the differences in spindle microtubule density. Scale bar, 5 μm, α-tubulin, and ch-TOG were stained with mouse monoclonal anti-α-tubulin and rabbit polyclonal anti-ch-TOG, respectively. (**B**) The bar graph shows the quantification of α-tubulin/MT intensity at the centrosomes (two). (**C**) The bar graph shows the percentage of cells (cells with above the average centrosomal microtubule intensity of TACC3 WT-expressed cells) with increased microtubule density phenotype in both the mutant cases. (**D**) The bar graph shows the quantification of ch-TOG intensity at the centrosomes (**E**) The bar graph shows the quantification of GFP-TACC3 intensity at the centrosomes. (**F**) The bar graph shows the quantification of ch-TOG intensity on the mitotic spindles. The regions of interest (ROI) used for quantification are shown in panel (A). (**G**) The bar graph shows the percentage of cells (cells with above the average ch-TOG intensity of TACC3 WT-expressed cells) with decreased ch-TOG intensity at the centrosomes phenotype in both the mutant cases. (**H**) The bar graph shows the percentage of cells (cells with above the average ch-TOG intensity of TACC3 WT-expressed cells) with increased ch-TOG intensity on the spindles in both the mutant cases. The bars represent mean ± S.E. The number of mitotic cells counted = 90–100 each (four independent experiments). ****, *P*<0.0001, ***, *P*< 0.001, **, *P*<0.01, *, *P*<0.05.

As perturbation of TACC3–ch-TOG interaction increased centrosomal recruitment of the γ-TuRC proteins, we sought to examine its effect on spindle microtubule assembly in mitotic cells. GFP-TACC3 (Δ678–681)-TACC3 shRNA, GFP-TACC3 (Δ682–688)-TACC3 shRNA, and GFP-TACC3 (WT)-TACC3 shRNA were expressed in HeLa Kyoto cells, and the microtubules of the mitotic synchronized metaphase cells were imaged. The spindle microtubules appeared to be abnormally dense in either of the TACC3 deletion mutant-expressed cells as compared with those of the TACC3-WT cells (Figure 2A). Intensity analysis showed ∼30% increase microtubule density (intensity) around the centrosomes in the mutant conditions compared with the WT (Figure 2B). About 75% of cells displayed increased microtubule density phenotype in both the mutant cases (Figure 2C). It has been shown previously that inhibition of TACC3–ch-TOG interaction affects ch-TOG localization on the spindle microtubules [24]. Consistently, we observed a diffused ch-TOG localization on the mitotic spindles in the TACC3 deletion mutant cells. Additionally, ch-TOG localization at the centrosomes in the TACC3 mutants cells also showed a moderate decrease as compared with TACC3-WT cells, which showed more intense ch-TOG localization at the mitotic centrosome and its close vicinity (Figures 1D and 2A). Intensity analysis of a small region around the centrosomes showed 20–25% reduction of ch-TOG intensity at the centrosomes in both the deletion mutants as compared with TACC3-WT-expressed cells (Figure 2D) and the change was statistically significant. The average expression levels of the GFP-tagged TACC3 proteins at the centrosomes did not change significantly in WT versus the TACC3 mutant cases (Figure 2E). Though the images of individual cells sometimes showed slight variations of GFP levels visibly, which happened to be observed both for TACC3-WT and TACC3 mutants conditions, but the increased microtubule density was apparent in majority of the mutants-expressed cells. Intensity analysis for ch-TOG on the mitotic spindles near to the chromosomes/metaphase plate (as shown in Figure 2A, top panel) showed ∼40% increase in ch-TOG intensity on the spindle microtubules in the mutant conditions compared with TACC3 WT cells (Figure 2F). The percentage of cells with reduced ch-TOG localization at the centrosomes and increased ch-TOG localization on the spindles was pronounced significantly in the TACC3-mutant’s expressed cells as compared with TACC3-WT condition (Figure 2G,H). Thus, the results indicate that TACC3–ch-TOG binding perturbation-mediated centrosomal γ-TuRC recruitment aggravates microtubule assembly at the mitotic centrosomes.

### Inhibition of TACC3–ch-TOG binding enhances the interaction of TACC3 with γ-TuRCs

**Figure 3:**
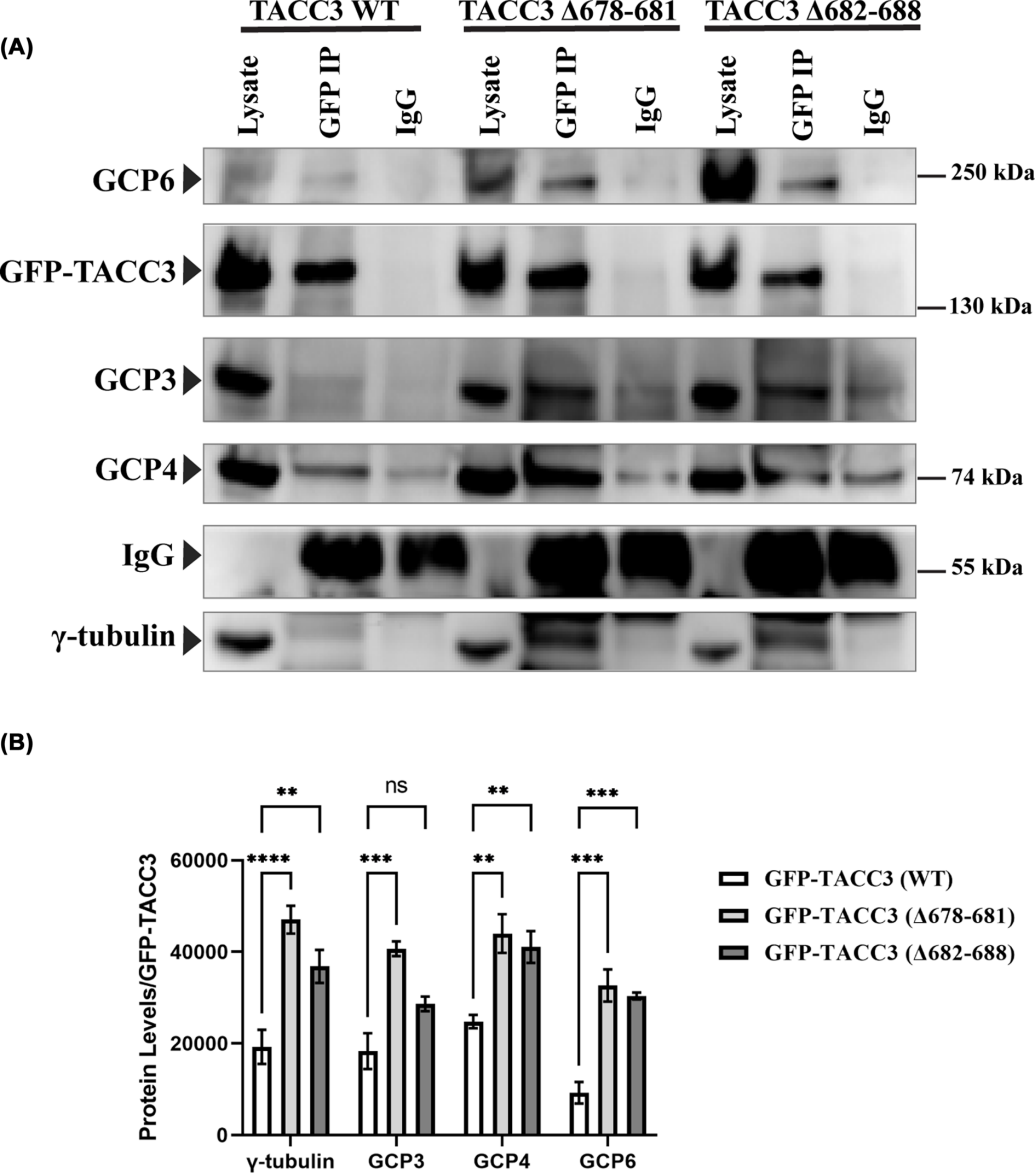
Inhibition of TACC3–ch-TOG binding enhances interaction of TACC3 with γ-TuRCs (**A**) Immunoprecipitation of GFP-tagged TACC3 proteins using GFP antibody in lysates of HeLa Kyoto cells transfected with GFP-TACC3 (WT)-TACC3 shRNA, GFP-TACC3 (Δ678–681)-TACC3 shRNA, or GFP-TACC3 (Δ682–688)-TACC3 shRNA. The immunoblots were probed with GFP-TACC3 (WT), GFP-TACC3 (Δ678–681), and GFP-TACC3 (Δ682–688) forms along with γ-tubulin, GCP3, GCP4, and GCP6 by using the respective antibodies. (**B**) Fold change of different γ-TuRC proteins from the GFP-TACC3 immunoprecipitates of GFP-TACC3 WT, GFP-TACC3 (Δ678–681), or GFP-TACC3 (Δ682–688) expressed cells are plotted (based on three experiments in each). Data are mean ± S.E. ****, *P*<0.0001, ***, *P*<0.001, **, *P*<0.01, ns: not significant.

As the centrosomal localization of the γ-TuRC proteins was increased upon inhibition of TACC3–ch-TOG interaction, we next tested whether the interaction of TACC3 to the γ-TuRC proteins was also affected. Co-immunoprecipitation (co-IP) with anti-GFP antibody was performed with the lysates of mitotic synchronized HeLa Kyoto cells expressed with GFP-TACC3 (Δ678–681)-TACC3 shRNA, GFP-TACC3 (Δ682–688)-TACC3 shRNA, and GFP-TACC3(WT)-TACC3 shRNA, respectively. Immunoprecipitates of both the TACC3 deletion mutants-expressed cells showed increased association of γ-tubulin, GCP3, 4, and 6, respectively, as compared with those associated with the GFP-TACC3-WT immunoprecipitate (Figure 3A,B). Intensity analysis from the IP data showed that the TACC3 deletion mutants could associate with almost double the amount of the γ-TuRC proteins as compared with TACC3-WT (Figure 3B), suggesting that TACC3 interaction with the γ-TuRC proteins is controlled by TACC3–chTOG binding.

### Deletion of the TACC3 Cterminal TACC domain affects centrosomal γtubulin localization but not TACC3 γTuRC interaction

**Figure 4:**
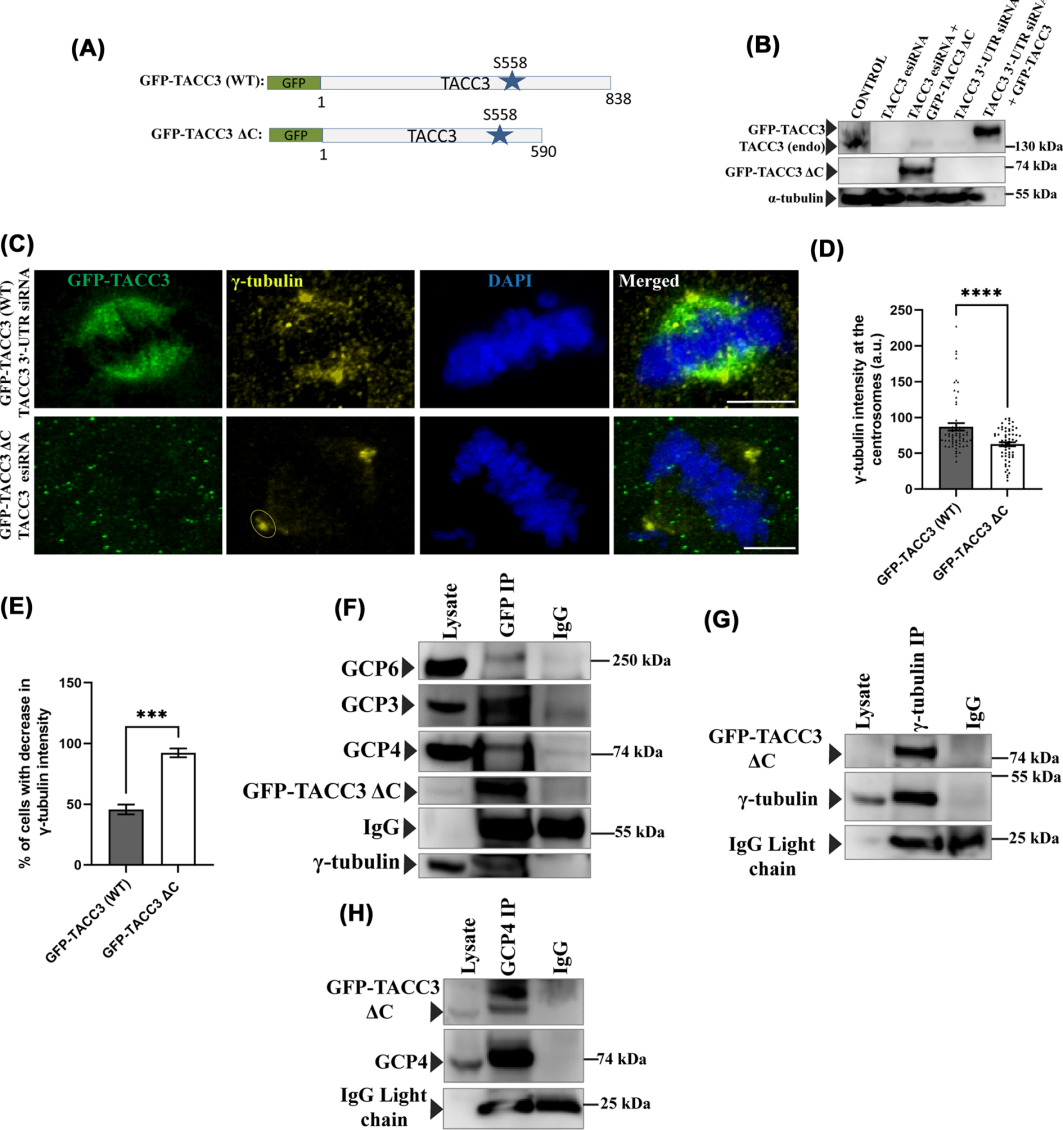
Deletion of TACC3 C-terminal (TACC) domain affects γ-tubulin localization but not TACC3–γ-TuRC interaction (**A**) Schematic representation of GFP-TACC3 (WT) and GFP-TACC3 ΔC (1–590) constructs. Position of Ser 558 phosphorylation site is also shown. (**B**) Lysates of HeLa Kyoto cells transfected with GFP-TACC3 (WT) along with TACC3 3′-UTR siRNA and GFP-TACC3 ΔC (1–590) along with TACC3 esiRNA for >48 h were analyzed by Western blot to detect the levels of exogenous TACC3 proteins and the depletion of endogenous TACC3 by the respective siRNAs. Both endogenous TACC3 and the exogenous TACC3 proteins were probed with mouse monoclonal anti-TACC3 antibody. α-tubulin was probed as a control. (**C**) Representative confocal images of GFP-TACC3 (WT) and GFP-TACC3 ΔC (1–590) transfected mitotic HeLa Kyoto cells showing the differences in γ-tubulin intensity. Scale bar, 5 μm, (**D**) The plots show γ-tubulin intensity at the centrosomes in different conditions as indicated. Centrosomal ROI used for quantification for both cases is shown. (**E**) The bar graph shows the percentage of cells (cells with above the average centrosomal γ-tubulin intensity of TACC3 WT-expressed cells) with decreased γ-tubulin intensity phenotype in GFP-TACC3 ΔC cells. The bars represent mean ± S.E. The number of mitotic cells counted = 60 each (four independent experiments). ****, *P*<0.0001, ***, *P*<0.001. (**F**) Immunoprecipitation of GFP-tagged TACC3 ΔC using the anti-GFP antibody in the lysate of HEK293T cells transfected with GFP-TACC3 ΔC. The immunoblots were probed with GFP-TACC3 ΔC, γ-tubulin, GCP3, GCP4, and GCP6 using the respective antibodies. (**G**) Immunoprecipitation of γ-tubulin using anti-γ-tubulin antibody in the lysate of HEK cells transfected with GFP-TACC3 ΔC. The immunoblots were probed with GFP-TACC3 ΔC and γ-tubulin using the respective antibodies. (**H**) Immunoprecipitation of GCP4 using anti-GCP4 antibody in the lysate of HEK293T cells transfected with GFP-TACC3 ΔC. The immunoblots were probed for GFP-TACC3 ΔC along with GCP4 by using respective antibodies.

Since the TACC3–ch-TOG interaction region is within the C-terminal TACC domain [638–838], and since the abrogation of TACC3–ch-TOG interaction intensifies TACC3–γ-TuRC interaction and induces centrosomal localization of γ-TuRC, we asked whether the TACC domain masks TACC3–γ-TuRC interaction and thereby limits γ-TuRC recruitment to the centrosomes. We checked the effect of deletion of the whole TACC domain by expressing the GFP tagged TACC3 (1–590) region on centrosomal localization of γ-TuRC. TACC3 (1–590) excluded the TACC domain, but included the Aurora A phosphorylation site on TACC3, Serine 558, which is involved in stabilizing TACC3–γ-TuRC interaction (Figure 4A) [18]. The results were compared with cells expressed with GFP-TACC3-WT. GFP-TACC3 (WT) full length and GFP-TACC3ΔC (1–590), referred as GFP-TACC3 ΔC constructs were expressed in HeLa Kyoto cells along with TACC3 3′-UTR siRNA and TACC3 esiRNA, respectively, to deplete the endogenous TACC3 upon their expression in cells [10]. Mitotic synchronized metaphase cells were collected after 48 h of transfection. Western blot analysis of the cell lysates showed the expression of the GFP-tagged TACC3 proteins with simultaneous depletion of endogenous TACC3 by the siRNAs was around 80–90% (Figure 4B). Previous studies showed that TACC domain is essential for TACC3 localization to the centrosome and the associated spindle microtubules [24,40]. Consistently, GFP-TACC3 ΔC localization appeared to be negligible at the centrosomes and the spindle microtubules (Figure 4C). Immunostaining of γ-tubulin revealed that in the GFP-TACC3ΔC-expressed metaphase cells, the centrosomal level of γ-tubulin was moderately reduced as compared with the GFP-TACC3 WT expressed condition (Figure 4C,D). About 90% of cells displayed the decreased γ-tubulin intensity phenotype in GFP-TACC3 ΔC cells (Figure 4E). Then, we assessed if the TACC3 variant devoid of the TACC domain can interact with the γ-TuRCs. We performed an exogenous GFP pull-down of GFP-TACC3 ΔC (1–590) expressed in HEK293T cells collected at the mitotic stage. Co-IP using GFP antibody from the GFP-TACC3 ΔC-expressed cells showed clear presence of the γ-TuRC proteins in the immunoprecipitate indicating that TACC3 (1–590) alone can interact with the γ-TuRC (Figure 4F). Further, the reverse pull-down of γ-tubulin and GCP4 also showed the presence of GFP-TACC3 ΔC (Figure 4G,H). Taken together, the results imply that though TACC3 (1–590) can interact with the γ-TuRC, the C-terminal TACC domain is essential for TACC3-mediated γ-TuRC recruitment to the centrosomes. This also suggests that TACC3–ch-TOG interaction is not likely to influence the TACC3–γ-TuRC interaction, but it affects the TACC-domain-mediated recruitment of the γ-TuRC to centrosomes.

### TACC3–ch-TOG interaction controls TACC3 phosphorylation

**Figure 5:**
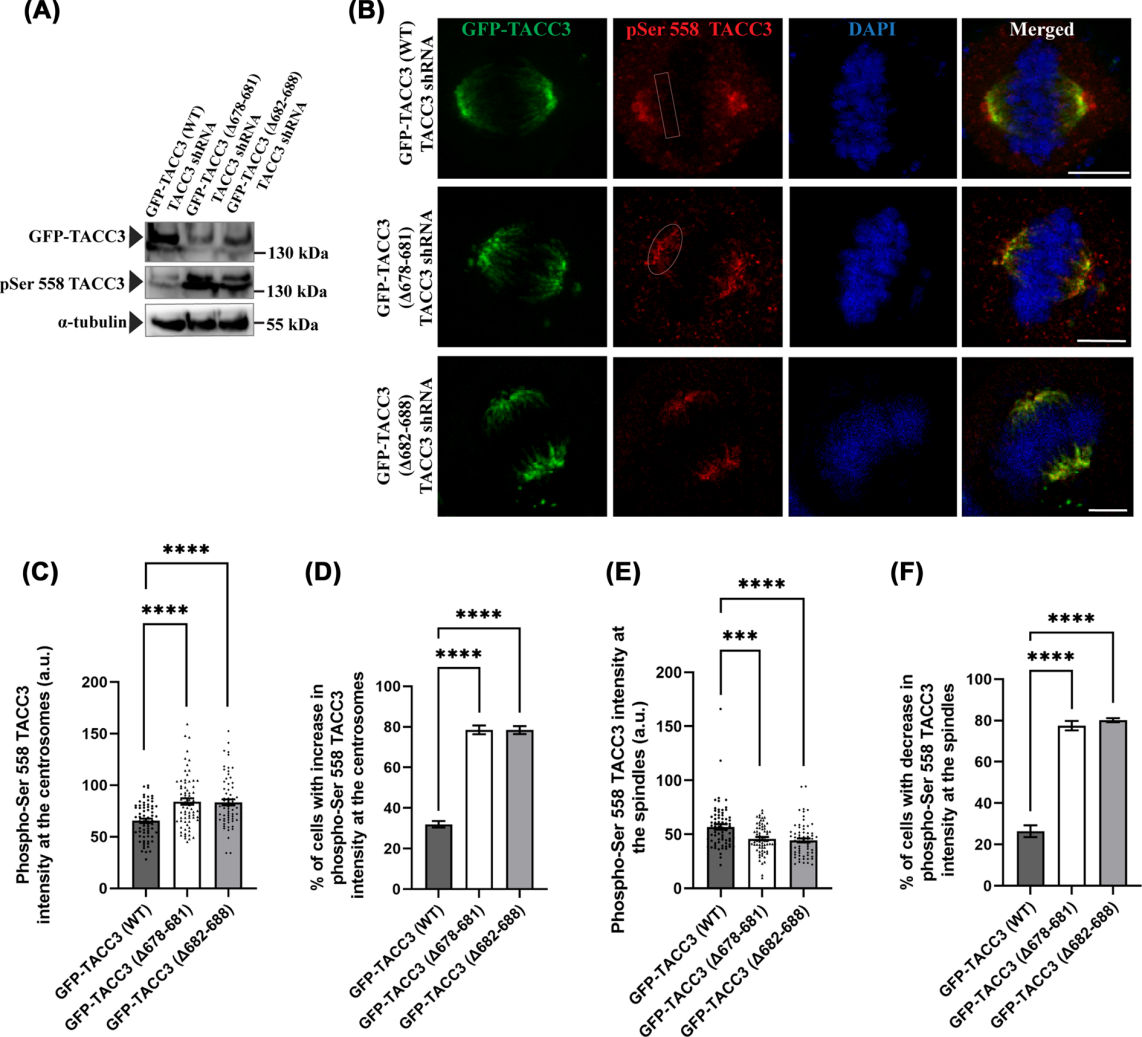
TACC3–ch-TOG interaction controls TACC3 phosphorylation (**A**) Lysates of HeLa Kyoto cells transfected with GFP-TACC3 (WT)-TACC3 shRNA, GFP-TACC3 (Δ682–688)-TACC3 shRNA, or GFP-TACC3 (Δ678–681)-TACC3 shRNA for 48 h were analyzed by Western blot to detect the levels of GFP-TACC3 proteins with simultaneously probing for endogenous TACC3. Both endogenous TACC3 and GFP-TACC3 proteins were probed with mouse monoclonal anti-TACC3 antibody. Phospho-Ser 558 TACC3 was detected using rabbit monoclonal anti-phospho-Ser 558 TACC3 antibody. α-tubulin was probed as a control. (**B**) Representative confocal images of GFP-TACC3 (WT)-TACC3 shRNA (top panel), GFP-TACC3 (Δ678–681)-TACC3 shRNA (middle panel), or GFP-TACC3 (Δ682–688)-TACC3 shRNA (bottom panel)-transfected mitotic HeLa Kyoto cells showing the localization of phospho-Ser 558 TACC3. Scale bar, 5 μm. Phospho-Ser 558 TACC3 was detected using rabbit monoclonal anti-phospho-Ser 558 TACC3 antibody. (**C**) The bar graph shows intensity of phospho-Ser 558 TACC3 at centrosomes in synchronized metaphase cells in ROI (19.47 μm^2^), as shown, around the centrosomes in different conditions as in panel (B). (**D**) The bar graph shows the percentage of cells (cells with above the average centrosomal phospho-Ser 558 TACC3 intensity of TACC3 WT-expressed cells) showing increased phospho-Ser 558 TACC3 intensity at the centrosomes in both the mutant cases. (**E**) The bar graph shows the quantification of phospho-Ser 558 TACC3 intensity on the mitotic spindles.The ROI used for quantification is shown in (b). (**F**) The bar graph shows the percentage of cells (cells with above the average phospho-Ser 558 TACC3 intensity of TACC3 WT-expressed cells) showing decreased phospho-Ser 558 TACC3 intensity on the spindles in both the mutant cases. The bars represent mean ± S.E. The number of cells counted = 70 each (four independent experiments) ****, *P*<0.0001, ***, *P*<0.001.

Previous studies have shown that phosphorylation at Ser 558 of TACC3 stabilizes TACC3 interaction with the γ-TuRC [18]. We, therefore, assessed whether disruption of ch-TOG binding to TACC3 affects TACC3 phosphorylation. GFP-TACC3 (Δ678–681)-TACC3 shRNA, GFP-TACC3 (Δ682–688)-TACC3 shRNA, and GFP-TACC3 (WT)-TACC3 shRNA were expressed in HeLa Kyoto cells, and the lysates of mitotic synchronized metaphase cells were examined for the level of phospho-Ser 558 TACC3. Phospho-Ser 558 TACC3 was immunostained using anti-phospho-Ser558-TACC3 antibody, which could be visualized using 4% paraformaldehyde fixation only. Interestingly, the levels of phospho-TACC3 in the cell lysates were visibly higher for both the TACC3 deletion mutants-expressed cells as compared with the TACC3-WT cells (Figure 5A). In the TACC3-WT cells, despite a high GFP-TACC3 total protein level, the amount the phosphorylated TACC3 was less; whereas the TACC3-deletion mutants cells showed increased levels of the phosphorylated TACC3, implying Ser 558 phosphorylation in TACC3 is stimulated in its ch-TOG unbound condition. We next inspected centrosomal localization of the Ser 558 phosphorylated TACC3 in the TACC3 deletion mutants conditions as compared with TACC3 WT-expressed cells. GFP-TACC3 (Δ678–681)-TACC3 shRNA, GFP-TACC3 (Δ682–688)-TACC3 shRNA-, and GFP-TACC3 (WT)-TACC3 shRNA-transfected HeLa Kyoto cells were analyzed for the localization pattern of phospho-Ser 558 TACC3 in synchronized metaphase cells by immunostaining for phospho-Ser 558 TACC3. Supporting the previous study, phospho-Ser 558 TACC3 was found to be localized predominantly at the centrosomes in the control metaphase cells (Figure 5B) [41]. The levels of phospho-Ser 558 TACC3 around the centrosomes (in ∼20 μm^2^ area around centrosome) appeared to increase significantly in the TACC3 deletion mutants-expressed cells as compared with the TACC3-WT cells (Figure 5C). Interestingly, phospho-Ser 558 TACC3 levels on the mitotic spindles showed a moderate (∼20%) decrease in the TACC3 deletion mutant cells as compared with TACC3 WT cells (Figure 5E). About 80% of cells displayed increased phospho-Ser 558 TACC3 intensity at the centrosomes and decreased phospho-Ser 558 TACC3 intensity on the spindles in both the mutant cases (Figure 5D,F). Intensity of phospho-TACC3 on the spindles was measured in selected rectangular regions (ROI) on the spindles away from the centrosomal area (Materials and Methods). The results suggest that TACC3–ch-TOG interaction affects phospho-TACC3 localization to the centrosomes.

### chTOG depletion does not significantly affect the level of γtubulin at the mitotic centrosomes

**Figure 6:**
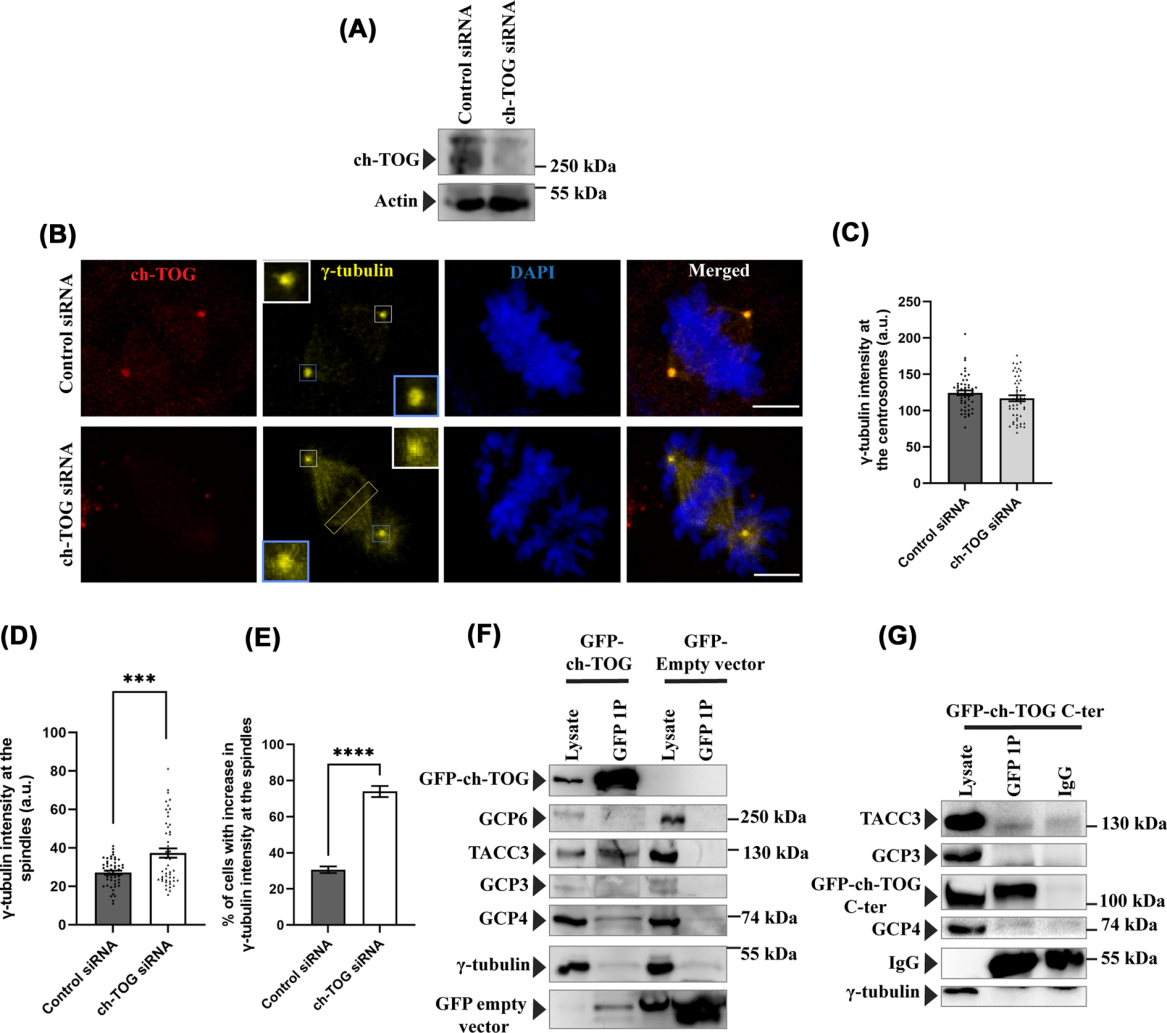
ch-TOG depletion does not significantly affect γ-tubulin localization at centrosomes (**A**) Lysates of HeLa Kyoto cells transfected with control siRNA and ch-TOG siRNA for 48 h were analyzed by Western blot to detect ch-TOG expression. (**B**) Representative confocal images of control siRNA or ch-TOG siRNA transfected mitotic Hela Kyoto cells showing the γ-tubulin intensity at the centrosomes and mitotic spindle. Scale bar, 5 μm. γ-tubulin was stained with mouse monoclonal anti-γ-tubulin antibody. Centrosomal regions are shown in an enlarged view. (**C**) The bar graph shows the quantification of centrosomal γ-tubulin intensity as of (b). The bars represent mean ± S.E. The number of cells counted = 50 each (four independent experiments). (**D**) The bar graph shows the quantification of γ-tubulin intensity on the spindles in different conditions as of (b). (**E**) The bar graph shows the percentage of cells (cells with above the average γ-tubulin intensity of TACC3 WT-expressed cells) showing increased γ-tubulin intensity at the spindles phenotype in ch-TOG siRNA treated cells. The bars represent mean ± S.E. The number of cells counted = 50 each (four independent experiments) ****, *P*<0.0001, ***, *P*<0.001. (**F**) Immunoprecipitation of GFP tagged ch-TOG using GFP coated beads (GFP-Trap) in the lysates of HeLa Kyoto cells stably expressing GFP-ch-TOG or control HeLa cells transfected with an empty GFP-vector (pcDNA3-EGFP). The immunoblots were probed for GFP-ch-TOG, γ-tubulin, GCP3, GCP4, GCP6, and TACC3 using the respective antibodies. (**G**) Immunoprecipitation of GFP-tagged ch-TOG C-terminus (1428–2032) using anti-GFP antibody in the lysates of HEK cells transfected with GFP-ch-TOG C-ter. The immunoblots were probed for GFP-ch-TOG, γ-tubulin, GCP3, GCP4, and TACC3 using the respective antibodies.

It has been demonstrated earlier that depletion of TACC3 results in significant reduction of γ-tubulin and other γ-TuRC proteins at the centrosomes [10,12,18]. A recent study showed that the C-terminal (1091–2065 aa) of XMAP215, the ch-TOG homolog in *Xenopus*, interacts with γ-tubulin *in vitro* [11]. Since abrogation of ch-TOG–TACC3 interaction affected centrosomal localization of the γ-TuRC proteins including γ-tubulin in human cells (Figure 1), we sought to investigate the effect of ch-TOG depletion on γ-tubulin localization at the mitotic centrosomes in human cells. HeLa Kyoto cells were transiently transfected with ch-TOG siRNA and the mitotic synchronized metaphase cells were assessed. chTOG was depleted efficiently (∼90%) by the ch-TOG siRNA (Figure 6A). Centrosomal localization of γ-tubulin was only marginally reduced (∼10%) in the ch-TOG depleted cells as compared with the control luciferase siRNA-treated cells (Figure 6B) and level of reduction was not statistically significant (Figure 6C). Since ch-TOG depletion also induces multipolar mitosis, only bipolar mitotic cells were quantified in this case for better comparison with the control cells. It was also observed that γ-tubulin was also localized on the mitotic spindles in ch-TOG-depleted cells and such phenomenon was not apparent in the control cells, where it was more concentrated at the centrosomes/spindle poles (Figure 6B). Intensity analysis of γ-tubulin on the mitotic spindles also supported the same (Figure 6D), suggesting that γ-tubulin diffuses from the centrosomes and gets accumulated on the mitotic spindles. Approximately 75% of cells displayed the increased γ-tubulin intensity on the spindles phenotype in ch-TOG siRNA treated cells (Figure 6E). To determine whether ch-TOG interacts with the γ-TuRC proteins, we performed a GFP pull down from GFP-ch-TOG- stably expressing HeLa cells by GFP-Trap agarose beads, and checked the association of the γ-TuRC proteins. To our surprise, γ-tubulin, GCP3, GCP4, and GCP6 were not found to associate with the GFP trap beads to detectable levels (Figure 6F). We further performed a GFP pull down from GFP-ch-TOG C-ter (1428–2032 aa) over-expressed cells by using anti-GFP antibody to probe for the association of the γ-TuRC proteins with ch-TOG C-terminus. GFP-ch-TOG C-ter (1428–2032 aa) did not show any detectable interaction with the γ-TuRC proteins (Figure 6G). However, as expected and it is known,TACC3 was found be associated with both full length GFP-ch-TOG and GFP-ch-TOG C-terminus immunoprecipitates under similar condition (Figure 6F,G) [24,38]. These results suggest that, in human cells, ch-TOG does not play an indispensable role in the localization of γ-tubulin to the mitotic centrosomes. Further, neither ch-TOG full-length protein nor its C-terminus interacts with the γ-TuRCs in human cells.

## Discussion

Optimal regulation of microtubule nucleation by the γ-TuRC requires association of several co-operating proteins as they seem to spatio-temporarily modulate its functions. Some of the co-operating proteins, such as CDK5RAP2 (human)/Spc72 (yeast), MZT 1/2A, [6,9,42] induce structural changes in the ring complex and thereby, activate its microtubule nucleation ability. There are also factors, such as NEDD1 that assists in recruiting the γ-TuRC to the centrosome [42,43]. Besides these classes, the proteins like TACC3, and XMAP215 also play important roles in γ-TuRC-mediated microtubule nucleation/assembly at and near the centrosomes either by imparting stabilization to the complex or by interacting with its components such as γ-tubulin [10,11,18]. However, molecular insights of such regulations needed more detailed analysis. Here, we have shown that abrogation of TACC3–ch-TOG interaction abnormally recruits more γ-TuRC proteins to the mitotic centrosomes resulting in aggravated mitotic spindle assembly (Figure 1). Earlier studies and also the results of this study (Figure 5) have shown that both the phosphorylation of TACC3 at Ser 558, which is located near to the TACC domain and the TACC domain itself are required for centrosomal recruitment of the γ-TuRC to the required level [18]. TACC3 phosphorylation at Ser 558 is also critical for stabilizing TACC3 interaction with the γ-TuRC as well as its assembly [18]. It is likely that the structural changes induced by phosphorylation provide the binding interface for γ-TuRC interaction. It is possible that such structural changes and the exposure of the γ-TuRC binding interface are inhibited, when ch-TOG remains bound to the coiled-coil domain located adjacent to the Ser 558 phosphorylation site of TACC3. Additionally, such structural hindrance could also influence the accessibility of the phosphorylation site by the targeting kinase, Aurora A. This possibility is feasible since ch-TOG exhibits an inverse relation with Aurora A with respect to its centrosomal localization [44]. Our results that the localization of phosphorylated TACC3 at the centrosomes is increased under ch-TOG unbound condition further support such possibility.

Suppression of TACC3–ch-TOG interaction induced ch-TOG removal from the mitotic centrosomes and its increased localization more in the spindle microtubule area. It has been shown previously that, ch-TOG localization on the spindle microtubules is increased, when it is complexed with TACC3 and clathrin; and the ch-TOG-TACC3–Clathrin complex induces spindle microtubule bundling [27,34,45]. The cells expressed with the ch-TOG non-binding mutants of TACC3 showed thick bundled spindle microtubules (Figure 2). Therefore, it is tempting to speculate that inhibition of TACC3 interaction with ch-TOG deregulates centrosomal localization of ch-TOG with its concomitant stabilization on the spindles. We also observed that the overall cellular level TACC3 phosphorylation at Ser 558 site was increased in its ch-TOG unbound condition (Figure 5). As centrosomal localization of γ-TuRC proteins is also stimulated under the same condition (Figure 1), it is supporting the notion that the elevated level of phospho-TACC3 imparts increased stabilization on the γ-TuRCs at the centrosomes and it is likely that ch-TOG is not involved in that process. Our data also showed that removal of the whole TACC domain does not seem to affect γ-TuRC interaction with TACC3 as TACC3 N-terminus (1–590), which consists of the Ser 558 phosphorylation site (Figure 4), binds to γ-TuRC efficiently as the full-length TACC3. Interestingly, in TACC3 1–590-expressed cells, centrosomal γ-tubulin localization was visibly impaired. It is possible that additional factors cooperate in γ-TuRC recruitment possibly by involving the TACC domain.

We found that depletion of ch-TOG did not affect centrosomal localization of γ-tubulin and further ch-TOG did not show any detectable interaction with the γ-TuRC proteins in human cells (Figure 6). The *Xenopus* form, XMAP215, however, stimulates microtubule assembly *ex vivo*, such as in *Xenopus* egg extract and further, it has been shown that the microtubule binding of the TOG domains is critical for this process. The same study also has shown that the recombinant form of XMAP215 domain consisting of the TOG5 followed by the C-terminus can bind to the purified γ-tubulin, though the same region fails to stimulate microtubule nucleation [11]. On the other hand, in human cells in this study, neither the full length ch-TOG nor its exclusive C-terminal domain (excluding of all the TOG 1–5 domains) exhibits interaction with the γ-TuRC (Figure 6). It suggests that the regulation of microtubule nucleation in *Xenopus* egg by XMAP215 is different than that by chTOG in human cells.

In conclusion, TACC3-ch-TOG binding puts a check on the centrosomal recruitment of the γ-TuRCs and controls the assembly of spindle microtubules from the mitotic centrosomes. TACC3 interaction with the γ-TuRCs and its centrosome-targeting phosphorylation levels are controlled by TACC3-ch-TOG binding and these processes also restrict the amount of ch-TOG to be associated with the spindles. The results unravel that TACC3-ch-TOG interaction assists in nucleating spindle microtubules to the required and optimal level and also regulates ch-TOG localization to the centrosome versus the spindle microtubules. Though the TACC3 phosphorylation site is away from the ch-TOG-interacting TACC domain, ch-TOG binding still controls TACC3 phosphorylation. It will be interesting to investigate in future the roles of the structural domains of ch-TOG that could control TACC3 phosphorylation and why such a process requires ch-TOG to be bound with TACC3.

## Methods

### Reagents and antibodies

Dulbecco’s modified Eagle’s medium (DMEM), fetal bovine serum (FBS), and antibiotic solutions were purchased from HiMedia, Inc. (Mumbai, India). Lipofectamine RNAiMAX, Lipofectamine 3000, and pre-stained protein ladder, 10 to 250 kDa were purchased from Thermo Fisher Scientific, U.S.A. PEI MAX- transfection agent from Polysciences. Mouse monoclonal anti-α-tubulin was purchased from Sigma (Cat # T5168); Mouse monoclonal anti-γ-tubulin for WB & IF (Cat # sc-17787), mouse monoclonal anti-TACC3 (Cat # sc-376883), mouse polyclonal anti-GCP4 (Cat # sc-271876), mouse monoclonal anti-GCP3 (Cat # sc-373758), anti-GCP6 (Cat # sc-374063), and mouse monoclonal anti-GFP antibody (Cat # sc-9996) were obtained from Santa Cruz Biotechnology, Inc.; rabbit polyclonal anti-ch-TOG antibody was obtained from Abcam (Cat # ab-236981); mouse monoclonal anti-actin was purchased from BD Biosciences (Cat # 612656); and rabbit monoclonal phospho-TACC3 antibody was purchased from Cell Signalling, U.S.A. (Cat # 8842). The secondary antibodies, anti-rabbit Alexa 568 and anti-mouse Cy5 were obtained from Jackson ImmunoResearch. DAPI was purchased from Sigma. GFP-Trap agarose beads used for IP were from Chromotech. Agarose A/G beads used for IP were from Santa Cruz Biotechnology, Inc.

### Cell culture and transfection

HeLa Kyoto cells were obtained from Dr Sachin Kotak, Indian Institute of Science (IISc), Bangalore (Originally provided by Daniel Gerlich, IMBA, Vienna). HEK 293 cells were from ATCC. ch-TOG-GFP stably expressing HeLa cells was a kind gift from Anthony Hyman lab (Max Planck Institute, Dresden, Germany). The cells were cultured in DMEM containing 10% FBS at 37°C under 5% CO_2_. GFP-TACC3 (WT)-TACC3 shRNA GFP-TACC3 (Δ678–681)-TACC3 shRNA and GFP-TACC3 (Δ682–688)-TACC3 shRNA constructs, cloned in p-Brain plasmid were from Addgene (Plasmid #69115 & #69116), deposited by Dr Stephen J. Royle, University of Warwick, UK. These constructs were used to express the deletion mutants of TACC3 and simultaneously deplete the endogenous TACC3 by TACC3 shRNA.

All esiRNAs were obtained from Sigma Aldrich (St. Louis, MO, U.S.A.). The esiRNA used was TACC3 esiRNA targeted against the 2166–2638 nucleotide region of TACC3 (NM_006342) Cat No. EHU063201 (Sigma) [10], ch-TOG esiRNA used was MISSION esiRNA (Sigma); and control firefly luciferase esiRNA, Cat No. EHUFLUC. All single siRNAs were purchased from Dharmacon (Lafayette, CO, U. S. A.). The sequence of the TACC3-3′-UTR-siRNA was UCUCUUAGGUGUCAUGUUC, and that of luciferase siRNA/control siRNA was GCCAUUCUAUCCUCUAGAGGAUG [10]. The full-length TACC3 DNA cloned into pCMV-6-AC (TACC3-GFP) with a C-terminal GFP tag, Cat No. RG210754 was obtained from Origene (MD, U.S.A.) [10]. TACC3 1-590 and ch-TOG 1428-2032 were cloned into a pcDNA3-EGFP empty vector obtained from Addgene (Plasmid #13031).

### Cell synchronization

Cell synchronization was performed as described earlier [46,47]. For synchronization of cells at mitotic metaphase, the cells were treated with thymidine (2 mM) after 12 h of transfection by plasmids for 18 h and then released for 9 h and again treated with thymidine for 17 h and after that, were collected after 10 h of thymidine release, the time at which cells reached mitosis. The metaphase mitotic cells were confirmed based on the chromosome alignment at the metaphase plate.

### Immunofluorescence microscopy

Cells fixed in methanol at −20°C were washed with phosphate-buffered saline (PBS), mixed with 2% bovine serum albumin and 0.5% Triton X-100 for blocking, and subsequently incubated with primary antibodies for 2 h. After washing, the cells were incubated with secondary antibodies and DAPI for 45 and 1 min, respectively. Coverslips were mounted using ProLong Gold anti-fade (Invitrogen), and images (63×) were captured using a Leica SP5 laser confocal microscope. Alternatively, cells fixed in 4% paraformaldehyde were washed with PBS, and primary antibodies were added and incubated overnight. After washing, the cells were incubated with secondary antibodies and DAPI for 1 h (1% BSA and 0.3% Triton X-100) and 1 min, respectively. The rest of the steps are identical, as mentioned above.

### Co-Immunoprecipitation (co-IP)

Cells were lysed with lysis buffer (4°C) containing 50 mM HEPES, pH 7.2, 0.1% Triton X-100, 100 mM NaCl, 1 mM EGTA, 1 mM MgCl_2_, phosphatase inhibitors 2 and 3, and a protease inhibitor mixture (Sigma) [46]. GFP protein was immunoprecipitated using mouse monoclonal antibody followed by the addition of protein A/G-agarose beads. GFP-coated beads (GFP-Trap) was used in the ch-TOG pull-down. The beads were washed with lysis buffer and then boiled in SDS-PAGE sample buffer for immunoblot analysis. Membranes were developed for immunoblot using the Immobilon reagent (Millipore), followed by imaging using ChemiDoc XRS System (Bio-Rad). Ser 558-phosphorylated TACC3 was probed by rabbit polyclonal phospho-TACC3 antibody (Cell Signaling, U.S.A.). For γ-tubulin and GCP4 IP, the same procedure was followed as above, using the respective antibodies.

### Image analysis

All immunofluorescence images were captured using a 63× (1.4 N.A.) oil immersion objective of a Leica SP5 confocal microscope. The same image acquisition and analysis settings were used for both control and treated conditions. The maximum intensity images were produced by projecting the images (Z-stacks) captured in three-dimensional optical sections at 0.25-μm intervals. The images were analyzed using Leica Application Suite Advanced Fluorescence Lite 2.8.0 software. Intensities were analyzed using Leica Application Suite Advanced Fluorescence Lite software. For analyzing the intensities of γ-tubulin and GCP6 at the spindle poles, the total intensity within an area spanning 14.95 μm^2^ and 1.7 μm^2^, respectively, around the spindle poles/centrosomes (sum of intensity at two poles) was quantified in each case (as shown in Figure 1). Similarly, for analyzing microtubule intensity around centrosomes, an area of 14.95 μm^2^ around centrosomes was taken (Figure 2). Area taken for measuring ch-TOG intensity around the centrosomes was 4.83 μm^2^ (Figure 2). ch-TOG at the spindles microtubules was measured by taking a rectangular area of 13.51 μm^2^ near the metaphase plate/chromosomes (Figure 2). γ-tubulin intensity around the centrosomes for Figure 4 was quantified by taking the area of 4.83 μm^2^. Phospho-Ser 558 TACC3 at the centrosomes was measured by taking 19.57 μm^2^ (Figure 5). Phospho-Ser 558 TACC3 at spindles microtubules was measured by taking a rectangular area of 14.4 μm^2^ near the metaphase plate (Figure 5). The γ-tubulin intensity in ch-TOG-depleted condition was measured by taking an area of 1.52 μm^2^ around the centrosomes (Figure 6). The γ-tubulin intensity in the same condition near the metaphase plate was quantified by using the area 13.71 μm^2^. Percentage of cellular phenotypes such as increased γ-tubulin and microtubule intensity, ch-TOG intensity at the centrosomes and on the spindles, decreased γ-tubulin intensity in TACC3 ΔC-expressed cells, Phospho-Ser 558 TACC3 levels at the centrosomes and spindles, and increased γ-tubulin intensity on the spindles in ch-TOG-depleted cells in TACC3 WT- versus TACC-mutants-expressed conditions were quantified based on the percentage of cells having above the average values of the respective parameters of TACC3 WT conditions in each case.

### Statistical analysis

The normality of the data was assessed using the Shapiro–Wilk test. The normally distributed data were analyzed with Student's *t*-test at the 99% confidence level. All data analyses were performed using Graphpad Prism. The data were plotted using Graphpad Prism. The figures were organized using Adobe Photoshop and Adobe Illustrator.

## Supplementary Material

Supplementary Data

## Data Availability

Data sharing is not applicable to the paper.

## Abbreviations

γ-TuRC: γ-tubulin ring complex
γ-TuSC: γ-tubulin small complex
DAPI: 4′,6-diamidino-2-phenylindole
DMEM: Dulbecco’s Modified Eagle Medium
EGTA: ethylene glycol-bis(β-aminoethyl ether)-N, N, N′, N′ tetraacetic acid
FBS: fetal bovine serum
GCP: γ-tubulin complex protein
HEPES: 4-(2-hydroxyethyl)-1-piperazineethanesulfonic acid
MTOC: microtubule-organizing center
PBS: phosphate-buffered saline
PCM: pericentriolar material
PEI: polyethylenimine
ROI: rectangular region
