# Supplementary material for: TACC3–ch-TOG interaction regulates spindle microtubule assembly by controlling centrosomal recruitment of γ-TuRC
Source: Biosci Rep. 2023 Mar 16;43(3):BSR20221882. doi: 10.1042/BSR20221882 (PMC10037420; doi:10.1042/BSR20221882)
Supplement: Supplementary Data [file BSR-2022-1882_supp.pdf]

Authorship Change Form

Please read the important information below before completing this form.

All sections should be completed in black ink, and all author names written in block capitals. This form can also be completed electronically.

Manuscript ID no.

BSR-2022-1882R1

Title of manuscript

TACC3-ch-TOG interaction regulates spindle microtubule assembly by controlling centrosomal recruitment of γ-TuRC

Important information:

Authors in Portland Press journals are defined as those who meet **all** criteria listed below:

(1) have made substantial contributions to conception and design, or acquisition of data, or analysis and interpretation of data;
(2) have drafted (written) the article or revised it for important intellectual content;
(3) have read and provided final approval of the version to be submitted/published;
(4) agree to be accountable for all aspects of the work in ensuring that questions related to the accuracy or integrity of any part of the work are appropriately investigated and resolved.

Our full Authorship policy is included at the end of this form. Please use this as a guide to completing this form. Once complete, please return this form (via email, please scan and email if completed offline) to the Editorial Office (editorial@portlandpress.com) to review. Based on the information provided, we will assess whether to approve your proposed change in authorship. Please note that we may contact your institution for more information or undertake a further investigation should we feel that this is needed in order for a final decision to be made.

A new authorship listing must be agreed by all authors (including those that have been removed or added) and approved by the Editorial Office before publication.

Assistance in writing the manuscript is not considered a significant contribution to warrant a change in authorship at this stage. In these instances, we will continue processing your manuscript with the original, accepted authorship. We suggest that you instead mention writing assistance in an updated Acknowledgements section.

In instances where you are not able to obtain agreement to change the authorship listing from all authors (including those to be removed), please refer to your institution(s) for investigation and inform us if you must do so. Portland Press will not mediate any authorship disputes.

If a fully completed form is not returned within 7 days of the date it was sent to the author, we will reject your authorship change request and proceed with publication using the original, accepted authorship listing.

Please provide the full authorship in the table below;

- **As per original submission (if change requested pre-acceptance)**

or

- **As per accepted manuscript (if change requested post-acceptance)**

	**First name(s)**	**Family name**
1st Author	Resmi	Rajeev
2nd Author	Suresh	Bhagyanath
3rd Author	Manu Rani S.	Devu Priya
4th Author	Swarnendu	Mukhopadhyay
5th Author	Tapas K.	Manna*
6th Author		
7th Author		
8th Author		

Please clearly mark the Corresponding Author with an Asterix (*). If your manuscript has more than 8 authors, please extend the table as required.

Please provide the requested change to authorship (full new authorship if requested changes were approved)

Please provide your new, requested full authorship in the order you would like it to be shown on your manuscript.

	**First name(s)**	**Family name**
1st Author	Resmi	Rajeev
2nd Author	Swarnendu	Mukhopadhyay
3rd Author	Suresh	Bhagyanath
4th Author	Manu Rani S.	Devu Priya
5th Author	Tapas K.	Manna*
6th Author		
7th Author		
8th Author		

Please clearly mark the Corresponding Author with an Asterix (*). If your manuscript has more than 8 authors, please extend the table as required.

Justification for change in authorship

In the box above, please provide us with your reasons for changing the authorship of your manuscript at this stage. If you are requesting the addition of authors, please explain why these authors were not included on the submitted manuscript and why they now merit authorship rather than being included in the Acknowledgements section. If you are removing authors, please explain what work was carried out by these individuals and consider if they need to be included in a revised acknowledgements section.

The author, Swarnendu Mukhopadhyay has carried out additional experiments asked by the reviewers for the revision work of the manuscript, which include carrying out several experiments for collecting additional data and analyze the data. Based on the relative contribution of all the authors in the revised manuscript, it is strongly felt that the author Swarnendu Mukhopadhyay now deserves authorship in the second position.

Author Contributions

If you are adding or removing authors, please provide a revised Author Contributions statement, ensuring that all authors in the new proposed authorship listing are mentioned.

Not applicable

Competing interests

This section can be left blank if there are no competing interests associated with your paper. If the new authorship results in a change in competing interests' status, please provide your new competing interests statement below.

Acknowledgement

This section can be left blank if your acknowledgement section is the same even though you have a different authorship list.

BIOCHEMICAL SOCIETY PORTLAND PRESS

Declaration of consent

This section must be signed by all authors regardless of status (unchanged, new and removed) for the authorship change request to be valid. (In lieu of an electronic signature, we will accept a handwritten signature (please print off the form, complete and sign and then scan and return); please note that the corresponding author takes responsibility that all persons listed on the paper are eligible for authorship).

Please delete addition/removal as appropriate. If you were included in OR are being deleted from the original authorship listing, please amend the text in red.

	First name	Family name		Signature	Affiliated institute	Date DD/MM/YYYY
1st Author	Resmi	Rajeev	I agree to the proposed authorship *to the authorship list.*	[illegible]		18/01/2023
2nd Author	Swarnendu	Mukhopadhyay	I agree to the proposed authorship *to the authorship list.*	[illegible]		18/01/2023
3rd Author	Suresh	Bhagyanath	I agree to the proposed authorship *to the authorship list.*	[illegible]		18/01/2023
4th Author	Manu Rani S.	Devu Priya	I agree to the proposed authorship *to the authorship list.*	[illegible]		18/01/2023
5th Author	Tapas K.	Manna	I agree to the proposed authorship *to the authorship list.*	[illegible]		18/01/23

Authorship policy:

Submission of a paper to the Portland Press journals implies that it has been approved by all of the named authors, that all persons entitled to authorship have been so named, that it reports unpublished work that is not under consideration for publication elsewhere in any language, that conflicts of interest are declared and that, if the paper is accepted for publication, the authors will grant the Biochemical Society an exclusive licence to publish the paper.

Authorship should be based on all the following criteria:

(1) substantial contributions to conception and design, or acquisition of data, or analysis and interpretation of data;
(2) drafting the article or revising it for important intellectual content;
(3) final approval of the version to be published;
(4) agreement to be accountable for all aspects of the work in ensuring that questions related to the accuracy or integrity of any part of the work are appropriately investigated and resolved.

Acquisition of funding, collection of data, or general supervision of the research group, alone, does not justify authorship. All contributors who do not meet the criteria for authorship should be listed in the Acknowledgements section. On submission, a paragraph outlining the contribution of each author to the study should be included and will be published as part of the paper. Please note that the author list that is provided at submission (and on the accepted paper) is what will be included on the published paper.

Changes to authorship

As a general rule, extensive changes to the authorship for an article are not permitted. During submission of the original and revised versions of the paper, the corresponding author takes responsibility that all persons listed on the paper should be authors (are eligible for authorship per the four criteria described above). Extensive changes to the author list will be investigated on a case-by-case basis, and if it cannot be ascertained why an extensive change has taken place, the paper may be rejected on that basis.

1. Authorship changes on submission of a revised version of a paper: Depending on the nature of the revisions asked for in decision letters sent to authors, new author(s) might be added to a revised version of a paper, particularly if new experimental work has been requested as part of the peer review process, and if the contributions of previously unnamed persons now merit authorship listing on the revised manuscript (criteria for authorship described above). In the event of the addition of authors at revision, the author contribution paragraph at the end of the manuscript must be updated to reflect what the newly added authors contributed to the paper.

If any authors are being removed from a revised paper, then it should be clear why that author has been removed, and written confirmation should be obtained from all authors (including the author who has been removed) confirming that they are aware of and agree with the removal of the author.

2. Authorship changes following acceptance of a paper and/or during the proof process: Accepted papers have been through peer review and at least one round of revision by the authors and have received a final “accept” decision from the Editor. Given this, we do not support addition or removal of authors post-acceptance.
